# Telomere and mitochondria mediated the association between dietary inflammatory index and mild cognitive impairment: A prospective cohort study

**DOI:** 10.1186/s12979-022-00326-4

**Published:** 2023-01-05

**Authors:** Qian Liu, Zhenshu Li, Ling Huang, Dezheng Zhou, Jingzhu Fu, Huilian Duan, Zehao Wang, Tong Yang, Jing Zhao, Wen Li, Huan Liu, Fei Ma, Changqing Sun, Guangshun Wang, Yue Du, Meilin Zhang, Yongjie Chen, Guowei Huang

**Affiliations:** 1 Department of Nutrition & Food Science, School of Public Health, Tianjin Medical University, Tianjin, China; 2 Tianjin Key Laboratory of Environment, Nutrition and Public Health, Tianjin, China; 3 Department of Epidemiology & Biostatistics, School of Public Health, Tianjin Medical University, Tianjin, China; 4 Neurosurgical Department of Baodi Clinical College, Tianjin Medical University, Tianjin, China; 5 Department of Tumor, Baodi Clinical College of Tianjin Medical University, Tianjin, China; 6 Department of Social Medicine and Health Management, School of Public Health, Tianjin Medical University, Tianjin, China

**Keywords:** Dietary inflammatory index, Mild cognitive impairment, Systemic immune inflammation index, System inflammation response index, Leukocyte telomere length, Mitochondrial DNA copy number

## Abstract

**Background:**

Diet and chronic inflammation might play a major role in the pathogenesis of mild cognitive impairment (MCI). In addition, peripheral blood leukocyte telomere length (LTL) and mitochondrial DNA copy number (mtDNAcn) might mediate the relationship between inflammation and MCI risk. The purpose of the present study is to evaluate whether inflammatory potential of diet assessed by dietary inflammatory index (DII), chronic inflammation, peripheral blood LTL, and mtDNAcn were associated with the risk of MCI.

**Results:**

A population-based cohort study was conducted with a total of 2944 participants. During a median follow-up of 2 years, 438 (14.90%) individuals were new-onset MCI. After adjustment, a higher score of DII (hazard ratio [*HR*]: 1.056, *95% CI*: 1.005, 1.109), a higher log systemic immune inflammation index (SII) (*HR*: 1.333, *95% CI*: 1.089, 1.633) and log system inflammation response index (SIRI) (*HR*: 1.487, *95% CI*: 1.024, 2.161) predicted elevated risk of MCI. An increased mtDNAcn (*HR*: 0.843, *95% CI*: 0.712, 0.997), but not LTL, predicted a decreased risk of MCI. Negative associations of log SII with LTL (*β*:-0.359, *95% CI*: -0.445, -0.273) and mtDNAcn (*β*:-0.048, *95% CI*: -0.090, -0.006) were found. Additionally, negative associations of log SIRI with LTL (*β*: -0.035, *95% CI*: -0.052, -0.017) and mtDNAcn (*β*:-0.136, *95% CI*: -0.216, -0.056) were also found. Path analysis suggested that SIRI, LTL, and mtDNAcn, in series, have mediation roles in the association between DII score and MCI risk.

**Conclusions:**

Higher DII, SII, and SIRI might predict a greater risk of MCI, while a longer LTL and an increased mtDNAcn were linked to a reduced risk of MCI among the older population. LTL and mtDNAcn could play mediation roles in the association between DII and MCI risk.

**Supplementary Information:**

The online version contains supplementary material available at 10.1186/s12979-022-00326-4.

## Background

Mild cognitive impairment (MCI) is an intermediate stage between normal cognition and dementia [1]. According to previous study, an estimated 10% to 15% of MCI patients convert to Alzheimer’s disease (AD) each year, whereas only 1% to 2% of older people with normal cognition have the same onset [1]. Therefore, early identification of individuals with MCI is beneficial to reduce future disease burden.

The progression of cognitive decline is a complex multifactorial degenerative process. In addition to genetics, the presences of vascular risk factors and environmental factors, such as lifestyle and dietary habits, are also associated with MCI development [2]. Among them, diet plays an important role in maintaining normal cognitive function and an increasing number of studies showed that diet affects cognition in multiple ways. In particular, inflammation has been suggested to mediate the relationship between diet and MCI [3]. For example, a diet rich in fruits and vegetables, which have high anti-inflammatory antioxidant functions, is linked to a lower risk of age-related cognitive decline [2]. On the other hand, western diet could lead to cognitive impairment due to an accelerated inflammation [4].

Dietary inflammatory index (DII) is a novel literature-derived and population-based index, and is designed to assess the anti- or pro-inflammatory potential of an individual’s diet [3]. A higher DII score indicates a higher inflammatory potential of diets. Previous studies showed that diets with a higher pro-inflammatory potential were linked to increased risks of MCI or dementia [3, 5]. Evidence from observational studies has demonstrated a significant association of DII with immunological parameter, including complement component C3, C-reactive protein (CRP), interleukin 6 (IL-6), tumor necrosis factor-α (TNF-α), white blood cell (WBC) counts, monocytes, neutrophils, and neutrophil to lymphocyte ratio (NLR) [6, 7]. Recently, two novel inflammatory markers, systemic immune inflammation index (SII) and system inflammation response index (SIRI), have been proposed to be integrated markers of systemic inflammation. The two indexes have been widely used to assess cancer or coronary artery disease [8, 9]. However, studies on the associations of diet, SII, and SIRI with MCI were limited.

Leukocyte telomere length (LTL) is an important biomarker of aging and a shortened LTL was associated with MCI [10]. Accelerated telomere attrition with age might be due to the increased inflammation level [11]. In addition to LTL, altered mitochondrial DNA copy number (mtDNAcn), an index of mitochondrial dysfunction, has been found to be positively related to LTL [12]. Meanwhile, several studies have indicated significant associations of mtDNAcn with MCI and AD [13]. However, few studies have tested whether LTL, mtDNAcn, or their interaction play a role in the relationship between inflammation and MCI.

In summary, the main aim of the present study is to investigate the longitudinal associations of inflammatory potential of diet, immune cells and their derived ratios (SII, and SIRI) with the risk of MCI. Besides, we hypothesized that LTL and mtDNAcn may mediate inflammation-induced MCI. Therefore, the underlying hypothesis was that a higher DII score may lead to increased inflammation levels, which in turn lead to accelerated telomere attrition and mitochondrial dysfunction. As a result, the risk of MCI would increase correspondingly.

## Results

### Characteristics of the study population

As shown in Table 1, MCI patients tended to be older, males, had lower total PA. At baseline and follow-up, incident MCI patients had significantly lower MMSE scores than non-MCI (all *P*-values < 0.05). MCI patients had a shorter LTL, a decreased mtDNAcn, and higher DII scores (all *P*-values < 0.05). There were no significant differences between the groups with regard to educational levels, BMI, alcohol drinking, smoking status, *APOE* ε4 carriers, history of hypertension, diabetes and hyperlipidemia, ADL scores, the count of platelet, neutrophil, lymphocyte, and monocyte, SII and SIRI scores (all *P*-values > 0.05).

**Table 1.** Baseline demographic and clinical characteristics of the participants (*n* = 2,944)

Characteristics	Incident MCI	Non-MCI	*P-*value
*n* (%)	438 (14.9)	2,506 (85.1)	
Age, years^*^	68.07 ± 5.18	67.37 ± 4.57	0.004
Female, *n* (%) ^‡^	220 (50.2)	1,420 (56.7)	0.012
Education level, *n* (%) ^‡^			0.971
Junior high school or below	356 (81.3)	2,035 (81.2)	
Senior high school and above	82 (18.7)	471 (18.8)	
BMI, kg/m^2*^	25.56 ± 3.24	25.84 ± 3.26	0.097
Drinking, *n* (%) ^‡^	103 (23.5)	572 (22.8)	0.751
Smoking status, *n* (%) ^‡^			0.257
Non-smoker	268 (61.2)	1,629 (65.0)	
Ex-smoker	39 (8.9)	184 (7.3)	
Current smoker	131 (29.9)	693 (27.7)	
Hypertension,* n* (%) ^‡^	177 (40.4)	1,091 (43.5)	0.223
Diabetes, *n* (%) ^‡^	56 (12.8)	358 (14.3)	0.405
Hyperlipidemia, *n* (%) ^‡^	33 (7.5)	206 (8.2)	0.628
Total PA, MET h/week^†^	32.00 (11.55, 65.10)	37.10 (13.81, 74.20)	0.030
*APOE ε4 n* (%) ^‡^	82 (18.7)	379 (15.1)	0.056
MMSE at baseline^*^	24.82±3.51	26.48±3.09	<0.001
MMSE at follow-up^*^	20.11 ± 3.67	25.41 ± 3.39	<0.001
ADL^*^	15.44 ± 2.88	15.91 ± 5.30	0.870
Platelet counts (×10^9^/L)^*^	204.87 ± 52.37	203.38 ± 54.11	0.595
Neutrophil counts (×10^9^/L)^*^	60.43 ± 7.72	60.02 ± 9.11	0.326
Lymphocyte counts (×10^9^/L)^*^	34.30 ± 7.73	34.37 ± 9.17	0.860
Monocyte counts (×10^9^/L)^†^	4.30 (3.60, 5.90)	4.40 (3.50, 6.40)	0.588
SII^†^	349.17 (266.05, 471.19)	354.31 (258.86, 470.17)	0.722
SIRI^†^	7.95 (6.21, 10.70)	7.98 (5.96, 11.66)	0.390
LTL^*^	1.31 ± 0.62	1.39 ± 0.72	0.048
mtDNAcn^*^	0.73 ± 0.57	0.81 ± 0.65	0.015
DII^†^	0.24 (-1.15, 1.82)	-0.09 (-1.53, 1.40)	0.005

Notes: *Data are presented as mean ± SD for independent-samples *t*-test; †data are presented as median (25th, 75th percentiles) for independent-samples *t*-test (after logarithmic transformation); ^‡^data are presented as *n* (%) for *chi*-square test. Monocyte counts, SII and SIRI were logarithmically transformed.

Abbreviations: MCI, mild cognitive impairment; BMI, body mass index; *PA*, physical activity; *APOE* ε4, apolipoprotein E polymorphism ε4; MMSE, Mini-Mental State Examination; ADL, activities of daily living; MET h/week, metabolic equivalent hours per week; SII, systematic inflammation index; SIRI, systematic inflammation response index; LTL, leukocyte telomere length; mtDNAcn, mitochondrial DNA copy number; DII, dietary inflammation index

### Longitudinal association between DII and incident MCI

The baseline demographic and clinical characteristics according to tertiles of DII are shown in Additional Table 1 and Additional Figure 1. Table 2 shows the longitudinal association between DII and the risk of MCI. In the crude model, compared to participants with the lowest DII tertile (T1), the risk for those with the highest DII tertile (T3) increased to 1.379 (*95%CI*: 1.093, 1.740) (*P* for trend = 0.007). After multivariable adjustment, the association between DII and MCI still remained significant (*P* for trend = 0.010). The results for the continuous variables were also statistically significant (*HR*: 1.056, *95%CI*: 1.005, 1.109).

**Fig 1.**
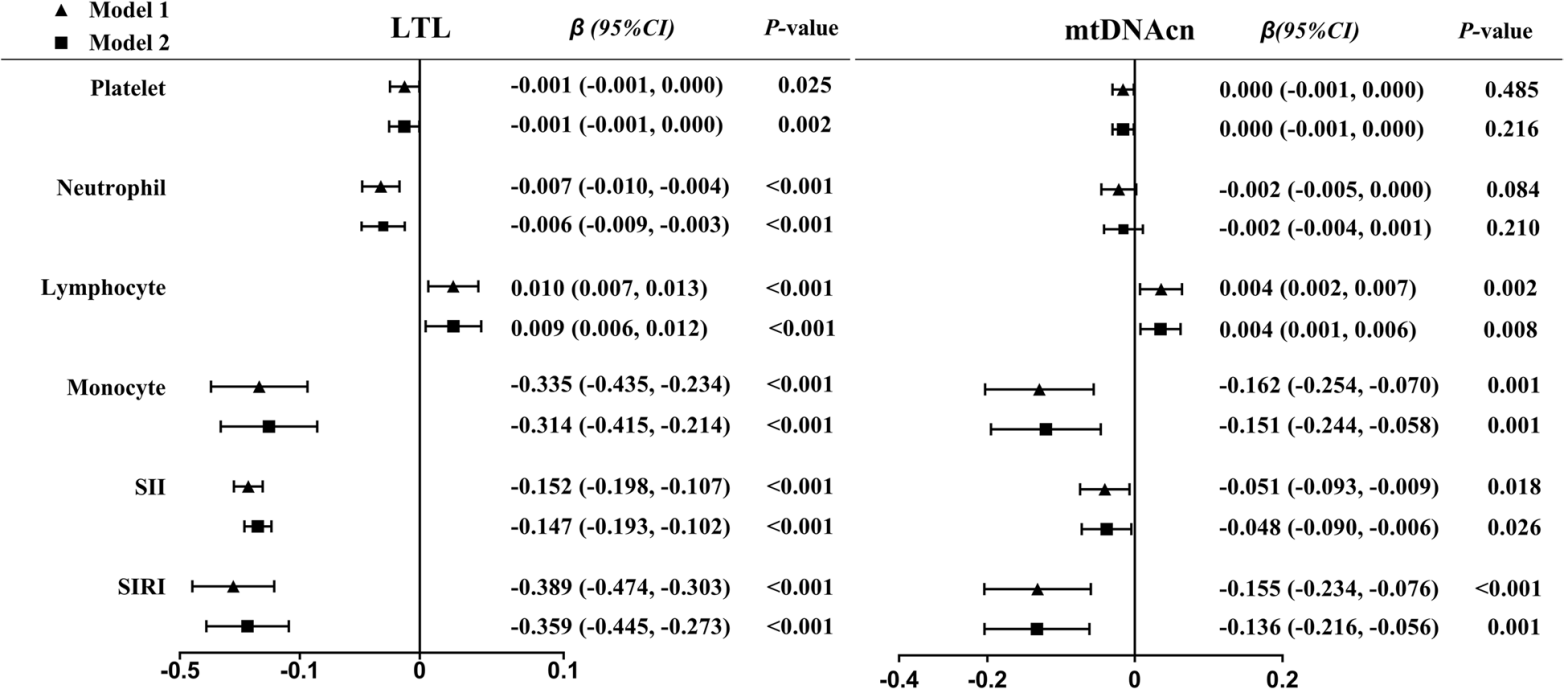
Associations of LTL and mtDNAcn with immunologic parameters (*n=*2,944). Analyses were conducted using linear regression analysis. Model 1 was crude model; model 2 adjusted for sex, age, body mass index, smoking, alcohol consumption, hypertension, diabetes, hyperlipidemia and total *PA*. Monocyte counts, SII and SIRI were logarithmically transformed. LTL, leukocyte telomere length; mtDNAcn, mitochondrial DNA copy number; SII, systematic inflammation index; SIRI, systematic inflammation response index

**Table 2.** Longitudinal associations between DII and MCI (*n=*2,944)

DII	MCIs/Non-MCI	Model 1	Model 2
		*HR (95%CI)*	*HR (95%CI)*
Tertiles of DII			
T1 (<-1.02)	121/861	1.00	1.00
T2 (-1.02~0.96)	146/734	1.193 (0.937, 1.517)	1.171 (0.919, 1.493)
T3 (>0.96)	171/811	1.379 (1.093, 1.740)	1.365 (1.077, 1.730)
*P* for trend		0.007	0.010
DII continuous	438/2,506	1.058 (1.008, 1.110)	1.056 (1.005, 1.109)

### Sensitivity analysis for the longitudinal association between DII and incident MCI

Based on the data from table 1, baseline MMSE score of the MCI group was significantly lower than the non-MCI group. Therefore, participants who converted to MCI might be already in the preclinical/prodromal stage when initially assessed. In order to assess the stability of the results, a sensitivity analysis was performed by excluding participants whose baseline MMSE score was at or below the 5th percentile (raw score of 20 or less). Results were consistent with main findings, and the results are displayed in Additional Table 2.

### Longitudinal associations between immunologic parameters, LTL and mtDNAcn with incident MCI

Table 3 shows the associations between immunologic parameters, LTL and mtDNAcn with incident MCI. In the crude model, a higher count of neutrophil (*HR*: 1.012, *95% CI*: 1.001, 1.024), a higher log SII (*HR*: 1.348, *95% CI*: 1.100, 1.651), a higher log SIRI (*HR*: 1.601, *95% CI*: 1.112, 2.304), a lower count of lymphocyte (*HR*: 0.985, *95% CI*: 0.973, 0.997), a longer LTL (*HR*: 0.851, *95% CI*: 0.739, 0.980) and an increased mtDNAcn (*HR*: 0.827, *95% CI*: 0.698, 0.979) were all significantly associated with an increased risk of MCI. After multivariate adjustment, similar results were observed except for the association of the count of neutrophil (*HR*: 1.010, *95% CI*: 0.998, 1.022) and LTL (*HR*: 0.877, *95% CI*: 0.761, 1.011) with MCI. The associations of DII and immunologic parameters are presented in the Additional table 3. The associations of DII and LTL, mtDNAcn are presented in the Additional table 4.

**Table 3.** Longitudinal associations between immunologic parameters, LTL and mtDNAcn with MCI (*n=*2,944)

Immunologic parameters	Model 1		Model 2	
	*HR (95%CI)*	*P-*value	*HR (95%CI)*	*P-*value
Platelet counts	1.001 (0.999, 1.003)	0.210	1.002 (1.000,1.003)	0.080
Neutrophil counts	1.012 (1.001, 1.024)	0.041	1.010 (0.998, 1.022)	0.094
Lymphocyte counts	0.985 (0.973, 0.997)	0.014	0.987 (0.975, 1.000)	0.045
Monocyte counts	1.353 (0.924, 1.982)	0.120	1.303 (0.887, 1.915)	0.178
SII	1.348 (1.100, 1.651)	0.004	1.333 (1.089, 1.633)	0.005
SIRI	1.601 (1.112, 2.304)	0.011	1.487 (1.024, 2.161)	0.037
LTL	0.851 (0.739, 0.980)	0.025	0.877 (0.761, 1.011)	0.070
mtDNAcn	0.827 (0.698, 0.979)	0.027	0.843 (0.712, 0.997)	0.046

Note: Analyses were conducted using *Cox* proportional hazard regression. Model 1 was crude model; model 2 adjusted for sex, age, body mass index, smoking, alcohol consumption, hypertension, diabetes, hyperlipidemia and total *PA* and *APOE* ε4. Monocyte counts, SII and SIRI were logarithmically transformed.

Abbreviations: MCI, mild cognitive impairment; *HR*, hazard ratio; *CI*, confidence interval; SII, systematic inflammation index; SIRI, systematic inflammation response index; LTL, leukocyte telomere length; mtDNAcn, mitochondrial DNA copy number

**Table 4.** Nested case-control analysis of the association between DII and MCI (*n*=153)

DII	MCIs/Non-MCI	Model 1	Model 2
		*OR (95%CI)*	*OR (95%CI)*
Tertiles of DII			
T1 (<-0.97)	11/39	1.00	1.00
T2 (-0.97~0.89)	18/34	2.002 (0.803, 4.987)	2.689 (1.022, 7.073)
T3 (>0.89)	22/29	3.548 (1.280, 9.831)	4.420 (1.432, 13.643)
*P* for trend		0.015	0.009
DII continuous	51/102	1.461 (1.155, 1.848)	1.622 (1.230, 2.139)

### Associations between LTL and mtDNAcn with immunologic parameters

The associations of LTL and mtDNAcn with immunologic parameters are shown in Figure 1. The associations of LTL with immunologic parameters were all statistically significant in both models (*P*-value < 0.05). Except for platelet and neutrophil counts, the associations of other immunologic parameters with mtDNAcn were found to be statistically significant (*P*-value < 0.05).

### Path analysis

**Fig 2.**
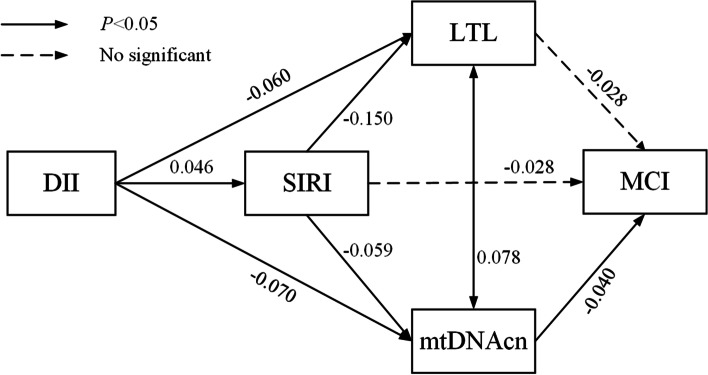
Path analysis for relationships between DII, SIRI, LTL, mtDNAcn and MCI. Standardized coefficients were reported in the figure. The model adjusted for sex, age, body mass index, smoking, alcohol consumption, hypertension, diabetes, hyperlipidemia, total PA and *APOE* ε4. SIRI was logarithmically transformed. Black solid lines represent *P-*value < 0.05 and black dashed lines represent *P-*value > 0.05. MCI, mild cognitive impairment; DII, dietary inflammation index; SIRI, systematic inflammation response index; LTL, leukocyte telomere length; mtDNAcn, mitochondrial DNA copy number

Based on the results above, path analysis was conducted to examine whether DII exerts its effect on MCI via postulated mediators (inflammation, LTL, and mtDNAcn), either singularly or in series. The results are shown in Figure 2. First, SIRI was a significant mediator of the associations of LTL and mtDNAcn with DII. In addition, mtDNAcn was a significant mediator of the relationship between DII and MCI*.* Second, path analysis supported the indirect path of an increased SIRI leading to a decreased mtDNAcn, which in turn lead to an increased MCI event. Another path showed that an increased DII lead to both decreased LTL and mtDNAcn, which in turn lead to an increased MCI event. These results suggested that SIRI, LTL, and mtDNAcn have mediation roles in the association between DII score and the risk of MCI (All *P*-values < 0.05). However, in this study, SII does not play a statistically significant mediating role.

### Sensitivity analysis

A nested case-control study was conducted based on the cohort study. All analyses were repeated using inflammation cytokines as indicators of inflammation instead of immune cells to validate the primary findings of cohort. Baseline characteristics are displayed in Additional table 5. The association between DII and the risk of MCI in conditional logistic regression is shown in Table 4. The result implied that a higher DII score was associated with a higher risk of MCI (*OR*: 1.622, *95%CI*: 1.230, 2.139), which was consistent with that of cohort study. The associations of DII, LTL and mtDNAcn with inflammatory cytokines are shown in Additional table 6 and Additional table 7.

**Table 5.** Nested case-control study on the associations of inflammatory cytokines, LTL, and mtDNAcn with MCI (*n*=153)

Biomarkers	Model 1		Model 2	
	*OR* (*95%CI*)	*P-*value	*OR* (*95%CI*)	*P-*value
IFN-γ	2.136 (1.413, 3.226)	<0.001	2.197 (1.401, 3.446)	0.001
IL-1β	1.503 (1.089, 2.074)	0.013	1.570 (1.099, 2.243)	0.013
IL-4	3.856 (1.068, 13.928)	0.039	4.335 (1.070, 17.565)	0.040
IL-6	3.091 (0.768, 12.437)	0.112	5.114 (1.091, 23.976)	0.038
IL-13	2.361 (1.443, 3.863)	0.001	2.314 (1.399, 3.827)	0.001
MCP-1	1.002 (0.999, 1.005)	0.223	1.001 (0.998, 1.005)	0.394
TNF-α	1.265 (1.130, 1.416)	<0.001	1.380 (1.186, 1.607)	<0.001
LTL	0.479 (0.248, 0.924)	0.028	0.421 (0.205, 0.868)	0.019
mtDNAcn	0.281 (0.120, 0.658)	0.003	0.269 (0.110, 0.656)	0.004

Note: Nested case-control analysis was performed as a sensitivity analysis. Model 1 was crude model; model 2 adjusted for sex, age, body mass index, smoking, alcohol consumption and *APOE* ε4. IFN-γ, IL-1β, IL-4, IL-6 and IL-13 were logarithmically transformed.

Abbreviation: MCI, mild cognitive impairment; *OR*, odds ratio; *CI*, confidence interval; IFN-γ, interferon-γ; IL, interleukin; MCP-1, monocyte chemoattractant protein-1; TNF-α, tumor necrosis factor-α; LTL, leukocyte telomere length; mtDNAcn, mitochondrial DNA copy number

The associations of inflammatory cytokines, LTL and mtDNAcn with MCI risk in sensitivity analysis are shown in Table 5. Higher IFN-γ (*OR*: 2.197, *95%CI*: 1.401, 3.446), IL-1β (*OR*: 1.570, *95%CI*: 1.099, 2.243), IL-4 (*OR*: 4.335, *95%CI*: 1.070, 17.565), IL-6 (*OR*: 5.114, *95%CI*: 1.091, 23.976), IL-13 (*OR*: 2.314, *95%CI*: 1.399, 3.827) and TNF-α (*OR*: 1.380, *95%CI*: 1.186, 1.607) levels were associated with an increased MCI risk, while a longer LTL (*OR*: 0.421, *95%CI*: 0.205, 0.868) and an increased mtDNAcn (*OR*: 0.269, *95%CI*: 0.110, 0.656) were associated with a decreased MCI risk.

## Discussion

In this longitudinal study, we found that a higher DII score was associated with a higher incidence of MCI. Moreover, a higher DII score was associated with SIRI, which indicated an increased inflammatory response. Furthermore, inflammation enhanced telomere shortening, as well as decreased mtDNAcn, which in turn were associated with a higher risk of MCI. In sensitivity analyses, the results were consistent with those of the cohort study.

Previous studies found that higher DII scores were associated with an increased risk of MCI or dementia [3, 5, 14]. For example, a cohort study reported that a diet with pro-inflammatory properties was associated with a worse cognitive function in later life and with higher risks of MCI or dementia among women [3]. Another study showed that higher DII scores at midlife were associated with a poorer cognitive function after 13 years [14]. The results of the present study were consistent with those studies mentioned above. The potential mechanisms might be explained in two aspects. First, diets may have an effect on altering risk for cardiovascular disease and metabolic control, which are major modifiable factors for chronic inflammation to further influence cognitive function. Second, an increased dietary inflammatory potential may directly increase the chronic inflammation, which has been associated with a decreased cognitive function [15].

In the present study, SII and SIRI were used as indicators of the system inflammatory response of body, and the relationships between DII, SII or SIRI and the risk of MCI were identified. Findings were consistent with previous studies that DII score was associated with systemic inflammation [16, 17]. A notable result of this study was that SII and SIRI were positively associated with the risk of MCI. Although there were few studies to examine the associations of SII and SIRI with MCI, the different components, including neutrophil, monocyte, lymphocyte, and platelet, have been widely discussed in MCI or AD. Previous studies have suggested that lymphocyte count was significantly lower in AD and MCI patients than that in controls [18]. In contrast to lymphocytes, the neutrophil [18], monocyte [19], and platelet [20] increased in MCI or AD patients. The relationship between SII and dementia has been investigated in a cohort study, and obtained similar results with the present study [21]. Recently, a short-term prospective cohort study in patients with COVID-19 reported that SII index could predict neurocognitive performance [22]. To the best of our knowledge, the relationship between SIRI and cognition has not been studied up to now. The definition of SIRI was calculated as neutrophil count*monocyte count/lymphocyte count. Namely, SIRI was the combination of NLR and monocyte count. Both NLR and monocyte counts have been demonstrated to be independent risk factors for cognitive decline [23, 24]. Therefore, SIRI index was biologically plausible to be associated with MCI.

Previous studies showed the association between DII and LTL in both cross-sectional and longitudinal studies, which suggested that diet might play an important role as a determinant of LTL through pro-inflammatory or anti-inflammatory mechanisms [25, 26]. Similarly, it has been reported that higher intakes of fruits and dietary flavanones, which have been identified with good anti-inflammatory effects, were associated with an increased leukocyte mtDNAcn [27]. Meanwhile, peripheral immunologic alterations could accelerate telomere attrition [28] and suppress mitochondrial function [29]. Dietary factors can affect LTL and mitochondrial function [25, 27]. Therefore, given the roles of telomere and mitochondrial function in the pathogenesis of MCI, we hypothesized that LTL and mtDNAcn could serve as intermediates between inflammation and MCI. The result supported by the path analysis was consistent with our hypothesis. This study evaluated the associations of LTL and mtDNAcn, alone or in combination, with the risk of MCI. In line with previous studies, the present study confirmed a major independent role of LTL in predicting the risk of MCI, which was consistent with previous finding that LTL was associated with cognitive decline [30, 31]. In addition, the result of path analyses in this study suggested that mtDNAcn was an important influencing factor for MCI rather than LTL. Mitochondria was principal hub in the regulation of immune and inflammatory responses [32]. A population-based study for obsessive-compulsive disorder, which was a chronic neuropsychiatric disorder, has reported that the disorder and an increased NLR were associated with mtDNAcn, rather than with LTL [33]. Furthermore, it has been suggested that inflammatory stress causes an increased reactive oxygen species production and accumulated mtDNA damage, and the interaction between oxidative stress and mitochondrial dysfunction can result in energetic failure and death, biological aging, and the pathogenesis of age-related diseases [32].

On the other hand, the path analysis also suggested that mitochondrial dysfunction and telomere erosion conjointly affected MCI pathogenesis. The result was consistent with the growing evidence of the strong molecular linkage between telomere and mitochondrial dysfunction [12, 34]. A previous study in community-dwelling older women suggested that telomere function may influence mitochondrial function [12]. Similarly, the combination of telomere length and mtDNAcn was useful for monitoring cognitive decline in the older adults aged over 75 years [34]. Therefore, the combination of telomere length and mtDNAcn may better predict disease development. Although the molecular mechanisms on linking LTL and mtDNAcn to MCI were rarely understood, several studies have suggested that telomere attrition has the ability to trigger the activation of p53, which can further lead to mitochondrial apoptosis and influence the risk of MCI [35].

Several strengths exist in the present study. First, the causal direction of the associations of inflammatory potential of dietary and systemic inflammation with MCI risk were ascertained in this prospective cohort. Second, to validate the cohort findings, a nested case-control study was further conducted as a sensitivity analysis by using the inflammatory cytokines instead of the complete blood counts. However, the limitations of this study need to be highlighted. First, information on anti-inflammatory medicine use was not collected, its impact cannot be corrected. Second, self-reported exposure data may be vulnerable to recall bias. Third, the mechanic explanation for these findings remains unclear, and the explanations given in the paper need to be further investigated. Additionally, a short follow-up period was also a limitation of this study.

## Conclusions

In this prospective cohort study, we found that higher DII, SII and SIRI were associated with an increased risk of MCI in the older adults. In addition, a shorter LTL and a decreased mtDNAcn were affected by elevated systemic inflammation, and could result in a higher risk of MCI. These data indicated that a higher DII score can promote development of MCI via systemic chronic inflammation-mediated exacerbation of telomere and mitochondrial dysfunction. Since SII and SIRI levels were readily available assessments in the laboratory, they may be proposed as cost-effective predictors for the incidence of MCI.

## Methods

### Study population

**Fig 3.**
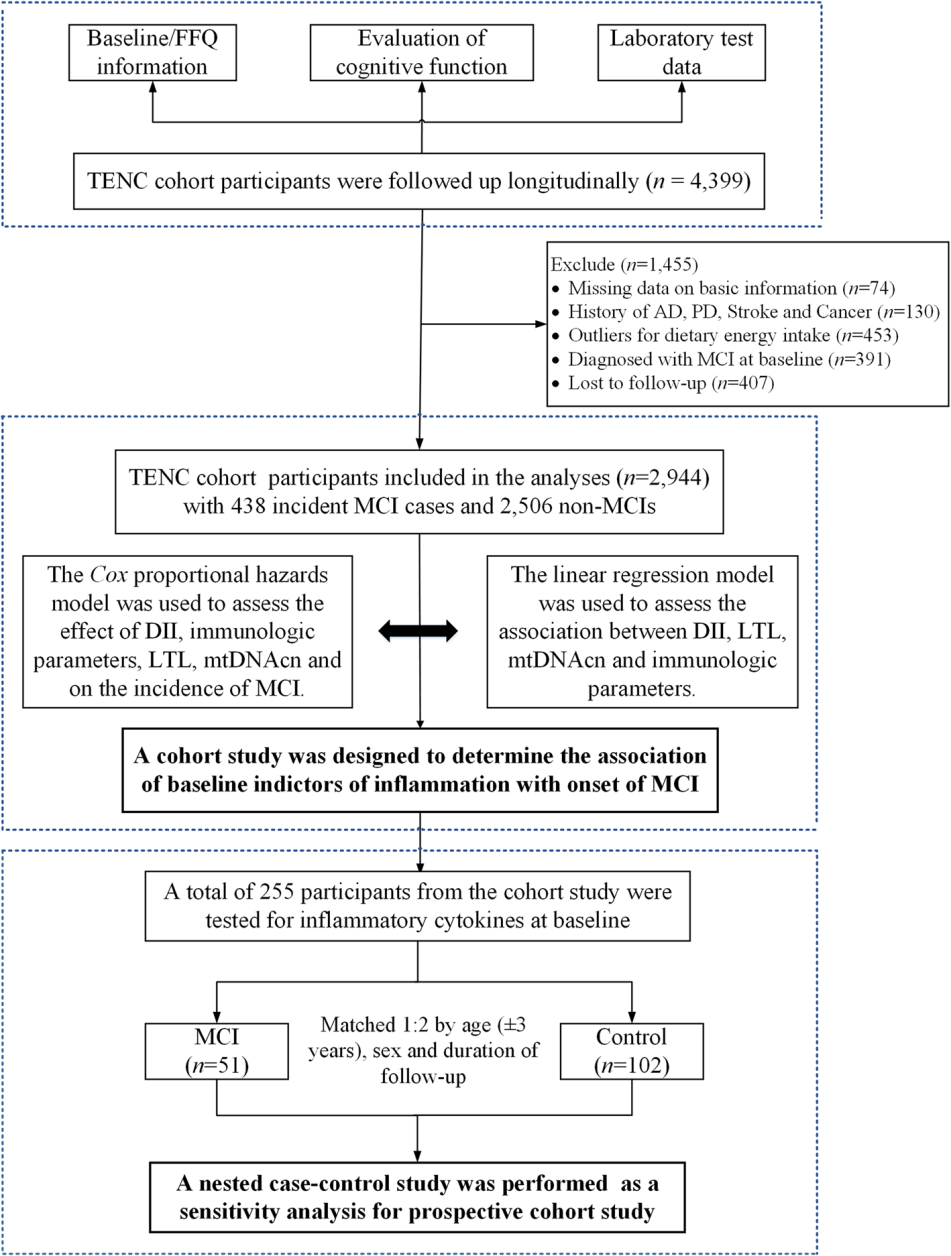
Flow diagram of the study process. Abbreviations: FFQ, food frequency questionnaires; PD, Parkinson's disease; AD, Alzheimer’s disease; MCI, mild cognitive impairment; DII, dietary inflammation index; LTL, leukocyte telomere length; mtDNAcn, mitochondrial DNA copy number

This study was a cohort study. Data were derived from the Tianjin Elderly Nutrition and Cognition (TENC) cohort study (registration identifier: ChiCTR2000034348). The TENC cohort study was conducted in the subjects aged 60 years or older and resided in the Baodi District of Tianjin, China. The sampling method in the TENC cohort study was multistage cluster sampling. In total, 5,577 eligible subjects from these communities were identified from March 2018 to June 2019. Those who were willing to participate were surveyed with a response rate of 88.60% (*n* = 4,933). All participants received a personal interview and a general physical examination by trained interviewers. The follow-up study was performed from March 2021 to June 2021. A total of 1,455 individuals were excluded from this analysis based on the following criteria: history of Parkinson's disease (PD), AD and stroke, which were clinically diagnosed by physician (*n* = 130); MCI at baseline (*n* = 391); missing demographic information (*n* = 74); abnormal total energy intakes (i.e., daily energy intake <800 kcal/day or >4800 kcal/day for male and <500 kcal/day or >4000 kcal/day for female, *n* = 453); and loss to follow-up (*n* = 407). Finally, a total of 2,944 individuals were included in this study (Figure 3). The study protocol was approved by the Ethics Committee of Tianjin Medical University (approval number: TMUhMEC2018013), and all participants provided informed consent before participation. All procedures in this study were conducted according to the Declaration of Helsinki.

### Definition of MCI

Diagnosis of MCI was based on the modified version of Petersen's criteria as previously described [36]: (i) subjective memory complaints over at least 6 months, preferably confirmed by an informant; (ii) Mini-Mental State Examination (MMSE) score of ≤ 17 points for illiterate people, ≤ 20 points for primary school level education, and ≤ 24 points for secondary education level and above; (iii) absence of dementia, AD, psychiatric disorders, cerebral damage or other physical diseases resulting in cognitive impairment; and (iv) little or no difficulty in activities of daily life, measured by the Activities of Daily Living Scale (ADL; <26 points). MCI patients had to meet all of these four criteria.

### Dietary assessment

The dietary assessment of participants was performed using a validated, simplified, and quantitative food frequency questionnaire (FFQ) that included 30 food items. Based on the FFQ, participants were required to recall their consumption frequencies and average amounts for the past 3 months. The frequency of each food item included 7 frequency categories as follows: (1) rarely eat or drink; (2) less than 1 time per week; (3) once a week; (4) 2-3 times per week; (5) 4-6 times per week; (6) once per day; and (7) twice or more per day. The consumption frequency of each food item was converted to daily intakes. According to the similarity of nutrient profiles and culinary usages among the foods and the grouping scheme used in other studies, we collapsed 30 food items into 20 predefined food groups. Information on frequency of intake and portion size were used to calculate the amount of each food item consumed on average, using the China Food Composition Table as the database.

### Calculation of DII score

The details of DII were available elsewhere [37]. Briefly, the dietary data for each study participant was first linked to the regionally representative global database of dietary surveys from 11 countries for each of the 45 parameters (i.e., foods, nutrients, and other food constituents). This global database provides a robust estimate of a mean and standard deviation for each of the food parameters considered. A z-score was derived by subtracting the “standard global mean” from the amount reported and then dividing this value by the standard deviation. This value was then converted to a centered percentile score, which was then multiplied by the respective food parameter inflammatory effect score to obtain the subject’s food parameter-specific DII score. All of the food parameter-specific DII scores were then summed to create the overall DII score for each participant. In the current study, DII score was calculated based on 24 available food parameters, which were as follows: energy, carbohydrate, protein, total fat, saturated fatty acids, monounsaturated fatty acids, polyunsaturated fatty acids, fiber, cholesterol, niacin, thiamine, riboflavin, folate, vitamin A, β-Carotene, vitamin C, vitamin E, iron, magnesium, selenium, zinc, isoflavones, alcohol, and anthocyanidins. To control the influence of total energy intake, we calculated the energy-adjusted version of the DII per 1000 calories of food consumed.

### Measurements of LTL and mtDNAcn

In the present study, LTL and mtDNAcn were determined using quantitative real-time polymerase chain reaction (q-PCR) method. The relative telomere length was calculated by the telomere to single-copy gene ratio. The mtDNAcn was calculated by the ND1 to single-copy gene ratio. The hemoglobin (HGB) gene was used as the reference gene (single-copy gene) for both LTL and mtDNAcn. All reactions were performed on a Roche LightCycler® 480 machine (Roche, Manheim, Germany) in the same lab. Each reaction was performed in triplicate and expressed as 2^(−ΔΔCt)^.

### Measurements of immunity markers

Blood samples were used to assay full blood count immediately after the blood draw. These measurements (including absolute counts of neutrophils, platelets lymphocytes, and monocytes) were performed using an Olympus AU5811 clinical chemistry analyzer (Tokyo, Japan) with standard laboratory techniques. The SII was defined as (P*N)/L and SIRI was defined as (N*M)/L, where N, M, and L represent the counts of peripheral neutrophil, monocyte, and lymphocyte, respectively. Enzyme-linked immunosorbent assay (ELISA) was applied to detect the inflammatory cytokines using MILLIPLEX®MAP Kit obtained from EMD Millipore Corporation (Billerica, MA).

### Genotyping of APOE ε4 allele

Genomic DNA was extracted from fasting venous EDTA blood using the QIAamp DNA Mini Kit (Spark Jade Science Co., Ltd, Shandong, China). Genotypes were determined via the Custom Taqman SNP Genotyping Assay by sequencing rs429358 (codon 112) and rs7412 (codon 158) at exon 4 of the *APOE* polymorphism, with the technical support of Shanghai OE Biotech Company.

### Covariates

Demographic and clinical characteristics as well as lifestyles were collected using a structured questionnaire. The demographic data included age, sex, educational levels, medical history, smoking status, alcohol consumption status. Physical activity (PA) was measured using a short version of the International Physical Activity Questionnaire (IPAQ), which reported as metabolic equivalent hours per day (METs-h/day) [38]. Body mass index (BMI) was measured by weight (kg)/height (m^2^). *APOE* polymorphism status was dichotomized as *APOE* ε4 carriers or non-carriers.

### Statistical analysis

Continuous variables were expressed as means ± SDs or medians (IQRs) based on the test of normality. Categorical variables were shown as frequencies (percentages). Normality of continuous variables was assessed using the *Skewness-Kurtosis* test. An independent-samples *t*-test was conducted for continuous variables and the *chi*-square test was used for categorical variables to compare the differences between those with and without MCI in all characteristics. Tertiles for DII were defined as follows: first tertile, less than -1.02 (T1); second tertile, -1.02~0.96 (T2); and third tertile, more than 0.96 (T3). *Cox* proportional hazard regression was performed to analyze the longitudinal associations of DII, immunologic parameters, LTL, and mtDNAcn with MCI. Results were presented as hazard ratios (*HRs*) and 95% confidence intervals (*CIs*). Linear regression analysis was performed to explore the relationships between DII, immunologic parameters, LTL, and mtDNAcn. Results were presented as *β*-coefficients with *95% CIs*. Model 1 was crude model; Model 2 adjusted for age, sex, BMI, smoking, alcohol consumption, history of hypertension, diabetes and hyperlipidemia, and total PA at baseline. Model 2 further adjusted for *APOE* ε4 carrier status when detecting MCI-related associations. Ordinal variables were not set to dummy variables, but as continuous variables to test the monotonic (upward or downward) trend (*P* for trend). The path analysis was performed using the procedures PROC REG and PROC CALIS from SAS 9.4. A nested case-control study was designed within the TENC cohort as a sensitivity analysis. For the nested case-control study, we matched up to two controls to each recurrent MCI patient following to the difference between case and control in age ≤ 3 years, and with the same sex and follow-up period. The nested case-control study repeated all analyses using inflammation cytokines as indicator of inflammation instead of immune cells to validate the primary findings of cohort. Paired *t*-test and conditional logistic regression were used to compare the differences between case and control in all characteristics and obtain odds ratio (*OR*) and *95% CI* of inflammation cytokines on MCI, respectively.

All analyses were performed using SPSS Version 25.0 (SPSS Inc., Chicago, IL, USA) and SAS version 9.4 (SAS Institute, Inc.). A two-sided *P* value< 0.05 was considered to be statistically significant.

## Supplementary Information

**Additional file 1.**

## Data Availability

The data that support the findings of this study are available from the corresponding author, Guowei Huang, upon reasonable request.

## Abbreviations

AD: Alzheimer’s disease
MCI: Mild cognitive impairment
BMI: Body mass index
PA: Physical activity
*APOE* ε4: Apolipoprotein E polymorphism ε4
MMSE: Mini-Mental State Examination
ADL: Activities of daily living
MET h/week: Metabolic equivalent hours per week
SII: Systematic inflammation index
SIRI: Systematic inflammation response index
LTL: Leukocyte telomere length
mtDNAcn: Mitochondrial DNA copy number
DII: Dietary inflammation index
