# Supplementary material for: Telomere and mitochondria mediated the association between dietary inflammatory index and mild cognitive impairment: A prospective cohort study
Source: Immun Ageing. 2023 Jan 5;20:1. doi: 10.1186/s12979-022-00326-4 (PMC9813461; doi:10.1186/s12979-022-00326-4)
Supplement: Supplementary file 1 — Additional file 1. [file 12979_2022_326_MOESM1_ESM.docx]

**Results**

**Characteristics of the study population across DII tertiles**

The baseline demographic and clinical characteristics according to tertiles of DII are shown in **Additional Table 1**. Participants in the second DII score group tended to be older (*P*-value = 0.046). Compared to individuals in the lowest tertile of DII score, individuals with a higher DII score had a lower BMI, tended to be current smokers, and had a decreased mtDNAcn (all *P*-values < 0.05). Compared to individuals in the lowest tertile of DII score, individuals with the highest DII score tend to be males, had a lower educational level, had a habit of drinking, had a lower total PA (all *P*-values < 0.05). In addition, in the highest DII group, individuals had higher levels of lymphocyte counts, monocyte counts, SII and SIRI, and shorter LTL (all *P*-values < 0.05). There were no significant differences in hypertension, diabetes, hyperlipidemia, *APOE* ε4 carriers, and other biochemical indicators across the DII tertiles (all *P*-values > 0.05).

| Additional Table 1. Baseline demographic and clinical characteristics according to tertiles of DII (*n*=2,944) | | | | |
| --- | --- | --- | --- | --- |
| Characteristics | T1 (*n=*982) | T2 (*n=*980) | T3 (*n=*982) | *P-*value |
| DII score | <-1.02 | -1.02~0.96 | >0.96 |  |
| Age, years^*^ | 67.21 ± 4.54 | 67.73 ± 4.64^a^ | 67.48 ± 4.81 | 0.046 |
| Female, *n* (%)^‡^ | 625 (63.6) | 530 (54.1) | 485 (49.4) | <0.001 |
| Education level, *n* (%)^‡^ |  |  |  | 0.038 |
| Junior high school or below | 777 (79.1) | 793 (80.9) | 821 (83.6)^a^ |  |
| Senior high school and above | 205 (20.9) | 187 (19.1) | 161 (16.4) |  |
| BMI (kg/m^2^)^*^ | 26.08 ± 3.36 | 25.72 ± 3.19^a^ | 25.60 ± 3.20^a^ | 0.004 |
| Drinking, *n* (%)^‡^ | 142 (14.5) | 226 (23.1) | 307 (31.3)^ab^ | <0.001 |
| Smoking status, *n* (%)^‡^ |  |  |  | <0.001 |
| Non-smoker | 701 (71.4) | 621 (63.4) | 575 (58.6) |  |
| Ex-smoker | 63 (6.4) | 87 (8.9) | 73 (7.4) |  |
| Current smoker | 218 (22.2) | 272 (27.8)^a^ | 334 (34.0)^ab^ |  |
| Hypertension, *n* (%)^‡^ | 409 (41.6) | 435 (44.4) | 424 (43.2) | 0.471 |
| Diabetes, *n* (%)^‡^ | 130 (13.2) | 146 (14.9) | 138 (14.1) | 0.572 |
| Hyperlipidemia, *n* (%)^‡^ | 95 (9.7) | 74 (7.6) | 70 (7.1)^a^ | 0.086 |
| Total PA, MET h/week^†^ | 39.55 (14.86, 67.71) | 37.10 (14.00, 74.20) | 28.00 (11.55, 75.21)^a^ | 0.068 |
| *APOE* *ε4* *n* (%)^‡^ | 157 (16.0) | 153 (15.6) | 151 (15.4) | 0.932 |
| Platelet counts (×10^9^/L)^*^ | 203.40 ± 54.61 | 201.61 ± 52.91 | 205.80 ± 53.99 | 0.224 |
| Neutrophil counts (×10^9^/L)^*^ | 59.62 ± 9.44 | 60.28 ± 8.90 | 60.36 ± 8.38 | 0.134 |
| Lymphocyte counts (×10^9^/L)^*^ | 34.89 ± 9.44 | 34.38 ± 9.00 | 33.80 ± 8.42^a^ | 0.027 |
| Monocyte counts (×10^9^/L)^†^ | 4.40 (3.50, 5.90) | 4.30 (3.50, 6.10) | 4.60 (3.60, 6.99)^ab^ | <0.001 |
| SII^†^ | 345.71 (245.57, 475.77) | 351.05 (258.11, 462.12) | 365.59 (277.04, 476.12)^a^ | 0.066 |
| SIRI^†^ | 7.65 (5.65, 10.85) | 7.72 (5.78, 11.77) | 8.35 (6.50, 11.92)^ab^ | <0.001 |
| LTL^*^ | 1.43 ± 0.72 | 1.41 ± 0.71 | 1.31 ± 0.68^ab^ | <0.001 |
| mtDNAcn^*^ | 0.87 ± 0.70 | 0.78 ± 0.61^a^ | 0.75 ± 0.60^a^ | <0.001 |
| Notes: *Data are presented as mean ± SD for independent-samples *t*-test; †data are presented as median (25th, 75th percentiles) for independent-samples *t*-test (after logarithmic transformation); ‡data are presented as *n* (%) for *chi*-square test. Monocyte counts, SII and SIRI were logarithmically transformed.  Abbreviations: DII, dietary inflammation index; MCI, mild cognitive impairment; BMI, body mass index; PA, physical activity; *APOE* ε4, apolipoprotein E polymorphism ε4; MMSE, Mini-Mental State Examination; ADL, activities of daily living; MET h/week, metabolic equivalent hours per week; SII, systematic inflammation index; SIRI, systematic inflammation response index; LTL, leukocyte telomere length; mtDNAcn, mitochondrial DNA copy number; T, tertiles. ^a^*P*<0.05, vs. T1；^b^*P*<0.05, vs. T2. | | | | |

**Additional Figure 1** demonstrates the incidence of MCI and MMSE score according to the tertiles of DII. Compared to participants in the lowest DII score, those with the highest DII score tended to have a higher incidence of MCI (17.40% vs 12.30%, *P*-value < 0.001) (**Additional Figure. 1A**). In addition, MMSE score in the highest tertile of DII (T3) was lower than those in lower tertiles (T1 and T2) (*P* for trend < 0.001) (**Additional Figure. 1B**).


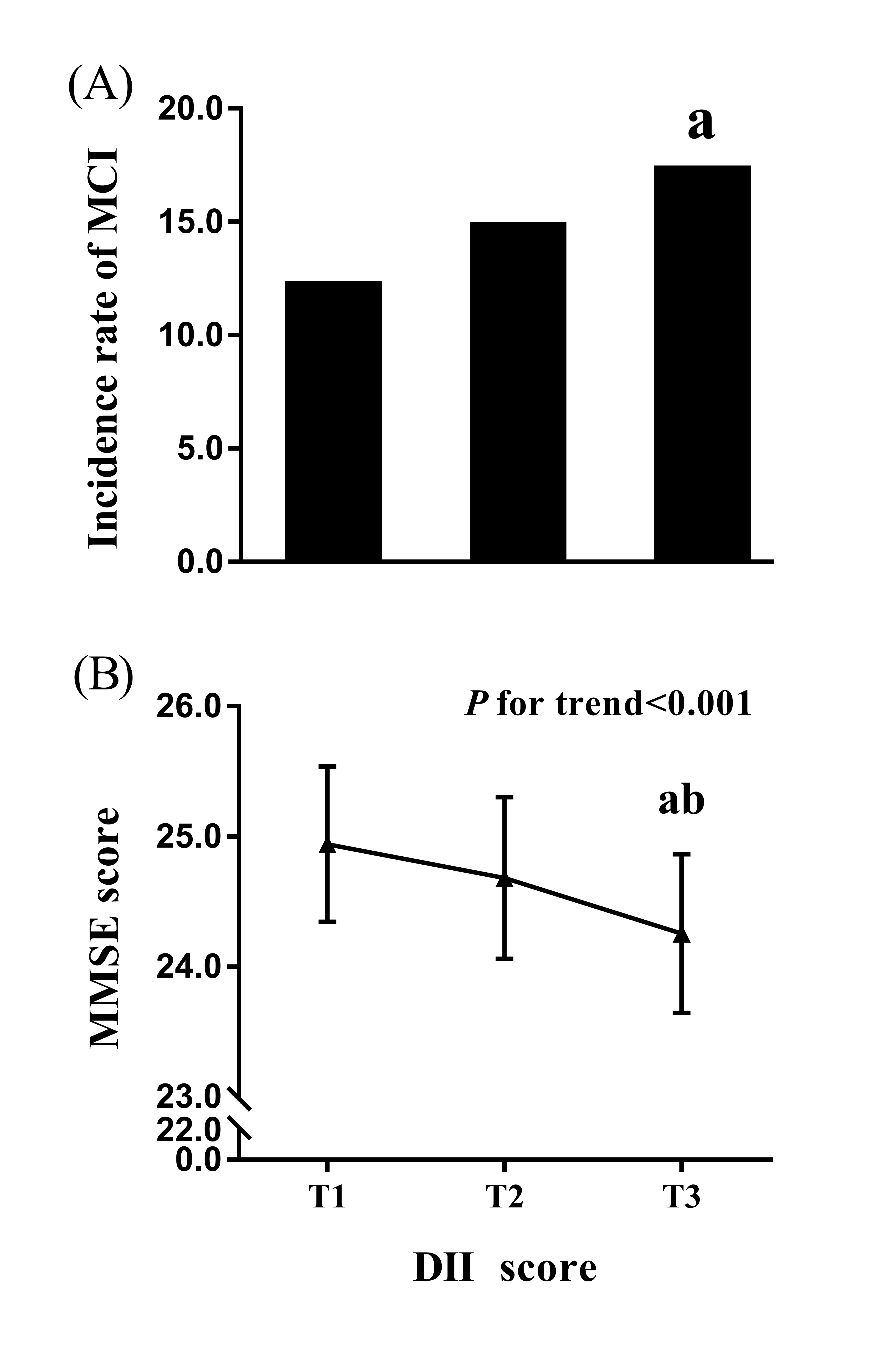


**Additional Figure 1.** The incidence of MCI and MMSE scores acorss DII tertiles. (A) The incidence of MCI (%). (B) MMSE score. ^a^: Compared to T1, *P*-value<0.05; ^b^: Compared to T2, *P*-value<0.05. The tertiles of DII score: T1: <-1.02; T2: -1.02~0.96; T3: >0.96. MCI, mild cognitive impairment; DII, dietary inflammatory index; MMSE, Mini-Mental State Examination; T, tertile.

“Results of sensitivity analysis in **Additional table 2** were consistent with main findings. In the crude model, compared to participants with the lowest DII tertile (T1), the risk for those with the highest DII tertile (T3) increased to 1.359 (*95%CI*: 1.061, 1.740) (*P* for trend = 0.014). The results were similar in adjusted model.”

| Additional Table 2. Sensitivity analysis of the longitudinal associations between DII and MCI in cohort study (*n=*2,763) | | | | | |
| --- | --- | --- | --- | --- | --- |
| DII | MCIs/Non-MCI |  | Model 1 |  | Model 2 |
|  |  |  | *HR (95%CI)* |  | *HR (95%CI)* |
| Tertiles of DII |  |  |  |  |  |
| T1 (<-1.04) | 107/814 |  | 1.00 |  | 1.00 |
| T2 (-1.04~0.94) | 126/794 |  | 1.145 (0.885, 1.482) |  | 1.112 (0.858, 1.441) |
| T3 (>0.94) | 151/771 |  | 1.359 (1.061, 1.740) |  | 1.313 (1.020, 1.690) |
| *P* for trend |  |  | 0.014 |  | 0.032 |
| Note: The sensitivity analysis was performed by excluding who may be in the preclinical/prodromal stage at baseline. Analyses were conducted using *Cox* proportional hazard regression. Model 1 was crude model; model 2 adjusted for sex, age, body mass index, smoking, alcohol consumption, hypertension, diabetes, hyperlipidemia, total *PA* and *APOE* ε4.  Abbreviations: MCI, mild cognitive impairment; DII, dietary inflammation index; *HR*, hazard ratio; *CI*, confidence interval; T, tertiles. | | | | | |

**Associations between DII and immunologic parameters**

**Additional table 3** displays the associations of DII and immunologic parameters. In the crude model, an increased DII score was associated with a decreased lymphocyte count (*β*: -0.200, *95% CI*: -0.367, -0.034). However, in the adjusted model, there was no significant association of DII score with lymphocyte count (*β*: -0.112, *95% CI*: -0.279, -0.056). The significant association between DII and monocyte counts was found in both crude (*β*: 0.008, *95% CI*: 0.003, 0.012) and adjusted (*β*: 0.007, *95% CI*: 0.002, 0.011) models. In addition, DII was positively associated with log SIRI in both crude (*β*: 0.010, *95% CI*: 0.004, 0.015) and adjusted (*β*: 0.007, *95% CI*: 0.001, 0.012) models.

| Additional Table 3. Associations of DII and immunologic parameters by linear regression analysis (*n=*2,944) | | | | | | | | | | | |
| --- | --- | --- | --- | --- | --- | --- | --- | --- | --- | --- | --- |
| Immunologic  parameters | Model | T1  (<-1.02) |  | T2 (-1.02~0.96) | |  | T3 (>0.96) | |  | DII continuous | |
|  |  |  |  | *β* (*95%CI)* | *P-*value |  | *β* (*95%CI)* | *P-*value |  | *β* (*95%CI)* | *P-*value |
| Platelet counts |  |  |  |  |  |  |  |  |  |  |  |
|  | Model 1 | 1.00 |  | -1.795 (-6.561, 2.972) | 0.460 |  | 2.394 (-2.370, 7.158) | 0.325 |  | 0.278 (-0.722, 1.278) | 0.585 |
|  | Model 2 | 1.00 |  | -0.059 (-4.918, 4.474) | 0.980 |  | 4.627 (-0.064, 9.411) | 0.056 |  | 0.803 (-0.193, 1.798) | 0.114 |
| Neutrophil counts |  |  |  |  |  |  |  |  |  |  |  |
|  | Model 1 | 1.00 |  | 0.656 (-0.133, 1.446) | 0.103 |  | 0.735 (-0.055, 1.524) | 0.068 |  | 0.161 (-0.005, 0.326) | 0.057 |
|  | Model 2 | 1.00 |  | 0.384 (-0.400, 1.168) | 0.337 |  | 0.415 (-0.377, 1.206) | 0.304 |  | 0.085 (-0.082, 0.251) | 0.319 |
| Lymphocyte counts |  |  |  |  |  |  |  |  |  |  |  |
|  | Model 1 | 1.00 |  | -0.504 (-1.298, 0.290) | 0.213 |  | -1.090 (-1.883, -0.296) | 0.007 |  | -0.200 (-0.367, -0.034) | 0.018 |
|  | Model 2 | 1.00 |  | -0.216 (-1.005, 0.572) | 0.591 |  | -0.721 (-1.517, 0.075) | 0.076 |  | -0.112 (-0.279, 0.056) | 0.191 |
| Monocyte counts |  |  |  |  |  |  |  |  |  |  |  |
|  | Model 1 | 1.00 |  | -0.002 (-0.024, 0.020) | 0.873 |  | 0.042 (0.020, 0.064) | <0.001 |  | 0.008 (0.003, 0.012) | 0.001 |
|  | Model 2 | 1.00 |  | -0.003 (-0.025, 0.019) | 0.768 |  | 0.038 (0.016, 0.060) | 0.001 |  | 0.007 (0.002, 0.011) | 0.005 |
| SII |  |  |  |  |  |  |  |  |  |  |  |
|  | Model 1 | 1.00 |  | 0.018 (-0.031, 0.067) | 0.468 |  | 0.057 (0.008, 0.106) | 0.023 |  | 0.010 (0.000, 0.020) | 0.061 |
|  | Model 2 | 1.00 |  | 0.013 (-0.036, 0.062) | 0.600 |  | 0.051 (0.001, 0.100) | 0.044 |  | 0.008 (-0.002, 0.019) | 0.117 |
| SIRI |  |  |  |  |  |  |  |  |  |  |  |
|  | Model 1 | 1.00 |  | 0.012 (-0.014, 0.038) | 0.372 |  | 0.053 (0.027, 0.079) | <0.001 |  | 0.010 (0.004, 0.015) | 0.001 |
|  | Model 2 | 1.00 |  | 0.004 (-0.022, 0.030) | 0.749 |  | 0.042 (0.016, 0.068) | 0.002 |  | 0.007 (0.001, 0.012) | 0.015 |
| Note: Analyses were conducted using linear regression analysis. Model 1 was crude model; model 2 adjusted for sex, age, body mass index, smoking, alcohol consumption, hypertension, diabetes, hyperlipidemia and total *PA*. Monocyte counts, SII and SIRI were logarithmically transformed.  Abbreviations: DII, dietary inflammation index; SII, systematic inflammation index; SIRI, systematic inflammation response index; *HR*, hazard ratio; *CI*, confidence interval; T, tertiles. | | | | | | | | | | | |

**Associations between DII, LTL and mtDNAcn**

**Additional Table 4** displays the associations between DII, LTL and mtDNAcn. After multivariate adjustment, an increased DII score was associated with a shorter LTL and a decreased mtDNAcn (*β*: -0.024, *95% CI*: -0.038, -0.011; and *β*: -0.024, *95% CI*: -0.036, -0.012, respectively).

| Additional Table 4. Associations of LTL and mtDNAcn with DII (*n=*2,944) | | | | | |
| --- | --- | --- | --- | --- | --- |
| Variables | Model 1 | |  | Model 2 | |
|  | *β* (*95%CI*) | *P* value |  | *β* (*95%CI*) | *P-*value |
| LTL | -0.029 (-0.042, -0.016) | <0.001 |  | -0.024 (-0.038, -0.011) | <0.001 |
| mtDNAcn | -0.028 (-0.040, -0.016) | <0.001 |  | -0.024 (-0.036, -0.012) | <0.001 |
| Note: Analyses were conducted using linear regression analysis. Model 1 was crude model; model 2 adjusted for sex, age, body mass index, smoking, alcohol consumption, hypertension, diabetes, hyperlipidemia, total PA and *APOE* ε4.  Abbreviations: DII, dietary inflammation index; LTL, leukocyte telomere length; mtDNAcn, mitochondrial DNA copy number. | | | | | |

Baseline characteristics of participants included in the nested case-control study are displayed in **Additional Table 5**. MCI patients tended to have a lower MMSE score, a shorter LTL, a decreased mtDNAcn, and higher DII scores (All *P*-value <0.05). There were no significant differences between two groups in other variables.

| Additional Table 5. Baseline characteristics of participants included in the nested case-control study (*n*=153) | | | |
| --- | --- | --- | --- |
| Characteristics | MCI | Control | *P-*value |
| *n* | 51 | 102 |  |
| Age, years* | 67.94 ± 4.87 | 68.02 ± 4.68 | 0.672 |
| Female, *n* (%)^‡^ | 35 (68.6) | 70 (68.6) | 1.000 |
| Education level, *n* (%)^‡^ |  |  | 0.556 |
| Junior high school or below | 30 (58.8) | 65 (63.7) |  |
| Senior high school and above | 21 (41.2) | 37 (36.3) |  |
| BMI (kg/m^2^)* | 25.21 ± 3.54 | 26.20 ± 3.28 | 0.075 |
| Drinking, *n* (%)^‡^ | 7 (13.7) | 21 (20.6) | 0.301 |
| Smoking status, *n* (%)^‡^ |  |  | 0.563 |
| Non-smoker | 34 (66.7) | 72 (70.6) |  |
| Ex-smoker | 3 (5.9) | 9 (8.8) |  |
| Current smoker | 14 (27.5) | 21 (20.6) |  |
| Hypertension, *n* (%)^‡^ | 18 (35.3) | 42 (41.2) | 0.482 |
| Diabetes, *n* (%)^‡^ | 6 (11.8) | 10 (9.8) | 0.709 |
| Hyperlipidemia, *n* (%)^‡^ | 4 (7.8) | 11 (10.8) | 0.564 |
| Total PA, MET h/week^†^ | 23.10 (11.50, 67.55) | 44.80 (23.10, 80.75) | 0.215 |
| *APOE* *ε4* *n* (%)^‡^ | 9 (17.6) | 16 (15.7) | 0.757 |
| MMSE* | 19.14 ± 3.80 | 25.81 ± 3.33 | <0.001 |
| ADL* | 15.31 ± 2.75 | 15.78 ± 5.09 | 0.480 |
| LTL* | 1.38 ± 0.64 | 1.14 ± 0.50 | 0.007 |
| mtDNAcn* | 0.97 ± 0.61 | 0.65 ± 0.44 | 0.001 |
| DII^†^ | 0.64 (-0.90, 2.51) | -0.27(-1.81, 1.06) | 0.001 |
| Notes: ^*^Data are presented as mean ± SD for independent-samples *t*-test; ^†^data are presented as median (25th, 75th percentiles) for independent-samples *t*-test (after logarithmic transformation); ^‡^data are presented as *n* (%) for *chi*-square test.  Abbreviations: DII, dietary inflammation index; MCI, mild cognitive impairment; BMI, body mass index; PA, physical activity; *APOE* ε4, apolipoprotein E polymorphism ε4; MMSE, Mini-Mental State Examination; ADL, activities of daily living; MET h/week, metabolic equivalent hours per week; SII, systematic inflammation index; SIRI, systematic inflammation response index; LTL, leukocyte telomere length; mtDNAcn, mitochondrial DNA copy number. | | | |

As shown in **Additional Table 6**, results of linear regression models found that DII score was positively associated with levels of IFN-γ (*β*: 0.518, *95% CI*: 0.053, 0.264), IL-4 (*β*: 0.030, *95% CI*: 0.003, 0.056), IL-6 (*β*: 0.022, *95% CI*: 0.002, 0.041), IL-13 (*β*: 0.102, *95% CI*: 0.014, 0.191), and TNF-α (*β*:1.315, *95% CI*: 0.307, 2.323), which implied a higher DII score was associated with elevated levels of inflammation.

The associations of LTL and mtDNAcn with inflammatory cytokines are shown in **Additional Table 7**. IL-13 (*β*: -0.097, *95%CI*: -0.191, -0.002) and TNF-α (*β*: -0.008, *95%CI*: -0.017, -0.000) were negatively related to LTL. Additionally, IFN-γ (*β*: -0.076, *95%CI*: -0.149, -0.002), IL-4 (*β*: -0.338, *95%CI*: -0.634, -0.043), IL-13 (*β*: -0.123, *95%CI*: -0.211, -0.035), and TNF-α (*β*: -0.012, *95%CI*: -0.020, -0.004) were negatively related to mtDNAcn.

| Additional Table 6. Nested case-control analyses of the association between DII and inflammatory cytokines (*n*=153) | | | | | |
| --- | --- | --- | --- | --- | --- |
| Inflammatory cytokines | Model 1 | |  | Model 2 | |
|  | *β* (*95%CI*) | *P-*value |  | *β* (*95%CI*) | *P-*value |
| IFN-γ | 0.153 (0.048, 0.258) | 0.005 |  | 0.158 (0.053, 0.264) | 0.004 |
| IL-1β | 0.096 (-0.011, 0.203) | 0.078 |  | 0.085 (-0.023, 0.192) | 0.122 |
| IL-4 | 0.029 (0.003, 0.055) | 0.028 |  | 0.030 (0.003, 0.056) | 0.027 |
| IL-6 | 0.024 (0.005, 0.043) | 0.015 |  | 0.022 (0.002, 0.041) | 0.030 |
| IL-13 | 0.091 (0.003, 0.179) | 0.042 |  | 0.102 (0.014, 0.191) | 0.023 |
| MCP-1 | -2.902 (-12.161, 6.357) | 0.537 |  | -3.585 (-13.089, 5.920) | 0.457 |
| TNF-α | 1.370 (0.389, 2.351) | 0.007 |  | 1.315 (0.307, 2.323) | 0.011 |
| Note: Nested case-control analysis was performed as a sensitivity analysis. Model 1 was crude model; model 2 adjusted for sex, age, body mass index, smoking and alcohol consumption. IFN-γ, IL-1β, IL-4, IL-6 and IL-13 were logarithmically transformed.  Abbreviation: DII, dietary inflammation index; *CI*, confidence interval; IFN-γ, interferon-γ; IL, interleukin; MCP-1, monocyte chemoattractant protein-1; TNF-α, tumor necrosis factor-α. | | | | | |

| Additional Table 7. Nested case-control analyses of the association between LTL and mtDNAcn with inflammatory cytokines (*n*=153) | | | | | |
| --- | --- | --- | --- | --- | --- |
| Variable | Model 1 | |  | Model 2 | |
|  | *β* (*95%CI*) | *P-*value |  | *β* (*95%CI*) | *P-*value |
| LTL |  |  |  |  |  |
| IFN-γ | -0.007 (-0.084, 0.069) | 0.855 |  | -0.009 (-0.088, 0.070) | 0.825 |
| IL-1β | -0.043 (-0.119, 0.033) | 0.263 |  | -0.046 (-0.124, 0.033) | 0.251 |
| IL-4 | 0.037 (-0.276, 0.350) | 0.817 |  | 0.041 (-0.277, 0.358) | 0.800 |
| IL-6 | -0.182 (-0.599, 0.235) | 0.390 |  | -0.193 (-0.625, 0.239) | 0.379 |
| IL-13 | -0.102 (-0.194, -0.011) | 0.028 |  | -0.097 (-0.191, -0.002) | 0.045 |
| MCP-1 | 0.000 (-0.001, 0.001) | 0.805 |  | 0.000 (-0.001, 0.001) | 0.849 |
| TNF-α | -0.008 (-0.016, 0.000) | 0.048 |  | -0.008 (-0.017, 0.000) | 0.043 |
| mtDNAcn |  |  |  |  |  |
| IFN-γ | -0.072 (-0.144, 0.000) | 0.051 |  | -0.076 (-0.149, -0.002) | 0.044 |
| IL-1*β* | 0.031 (-0.041, 0.104) | 0.396 |  | 0.020 (-0.055, 0.095) | 0.599 |
| IL-4 | -0.312 (-0.608, -0.017) | 0.038 |  | -0.338 (-0.634, -0.043) | 0.025 |
| IL-6 | -0.288 (-0.685, 0.109) | 0.154 |  | -0.308 (-0.715, 0.099) | 0.137 |
| IL-13 | -0.107 (-0.194, -0.020) | 0.016 |  | -0.123 (-0.211, -0.035) | 0.007 |
| MCP-1 | 0.000 (-0.001, 0.001) | 0.893 |  | 0.000 (-0.001, 0.001) | 0.876 |
| TNF-α | -0.012 (-0.020, -0.004) | 0.002 |  | -0.012 (-0.020, -0.004) | 0.002 |
| Note: Nested case-control analysis was performed as a sensitivity analysis. Model 1 was crude model; model 2 adjusted for sex, age, body mass index, smoking and alcohol drinking. IFN-γ, IL-1β, IL-4, IL-6 and IL-13 were logarithmically transformed.  Abbreviation: MCI, mild cognitive impairment; *CI*, confidence interval; IFN-γ, interferon-γ; IL, interleukin; MCP-1, monocyte chemoattractant protein-1; TNF-α, tumor necrosis factor-α; LTL, leukocyte telomere length; mtDNAcn, mitochondrial DNA copy number. | | | | | |
